# Living on the edge: Was demographic weakness the cause of Neanderthal demise?

**DOI:** 10.1371/journal.pone.0216742

**Published:** 2019-05-29

**Authors:** Anna Degioanni, Christophe Bonenfant, Sandrine Cabut, Silvana Condemi

**Affiliations:** 1 Aix Marseille Université, CNRS, Minist Culture, LAMPEA, Aix-en-Provence, France; 2 UMR CNRS Laboratoire Biométrie et Biologie Évolutive, Université Claude Bernard Lyon Villeurbanne, Villeurbanne, France; 3 Aix Marseille Université, CNRS, EFS, ADES, Marseille, France; Max Planck Institute for the Science of Human History, GERMANY

## Abstract

The causes of disappearance of the Neanderthals, the only human population living in Europe before the arrival of *Homo sapiens*, have been debated for decades by the scientific community. Different hypotheses have been advanced to explain this demise, such as cognitive, adaptive and cultural inferiority of Neanderthals. Here, we investigate the disappearance of Neanderthals by examining the extent of demographic changes needed over a period of 10,000 years (yrs) to lead to their extinction. In regard to such fossil populations, we inferred demographic parameters from present day and past hunter-gatherer populations, and from bio-anthropological rules. We used demographic modeling and simulations to identify the set of plausible demographic parameters of the Neanderthal population compatible with the observed dynamics, and to explore the circumstances under which they might have led to the disappearance of Neanderthals. A slight (<4%) but continuous decrease in the fertility rate of younger Neanderthal women could have had a significant impact on these dynamics, and could have precipitated their demise. Our results open the way to non-catastrophic events as plausible explanations for Neanderthal extinction.

## Introduction

The Neanderthals, a human metapopulation that lived between 250,000 and 40,000 yrs ago (OIS 7–3), is arguably the best known human fossil group. Since the discovery of the first Neanderthal specimens in 1856, their origin, evolution, differentiation, variability and genetics have been intensively studied. We have come to the understanding that the Neanderthals emerged from the European branch of *Homo heidelbergensis* [1–5] and that their differentiation in Europe has been the result of a long evolutionary process [6–8]. Neanderthals, who were the only humans on the European territory, disappeared during the OIS 3, when *Homo sapiens* arrived.

The causes of Neanderthal disappearance fueled a vigorous scientific debate and a number of hypotheses have been put forward to account for their demise (for a recent review see [9]). Because the Neanderthals disappeared at a time when *Homo sapiens* colonized Europe, their extinction has been related to the expansion of *Homo sapiens*. According to the most commonly accepted hypothesis, the Neanderthals would have competed with *Homo sapiens* for food resources and the replacement of Neanderthals would have been favored by *Homo sapiens'* greater technical skills [10,11], their greater cognitive abilities [11–15], Neanderthal’s narrower diet [16,17] and lower social capacities and network [11,18–21]. However, some prehistorians dispute the superior capacities of the first *Homo sapiens* in Europe compared to Neanderthals [20,22–27]. In light of our current knowledge about European colonization of the Americas in modern times, some authors have suggested that the disappearance of Neanderthals was also brought about by violent confrontations between the two populations [28,29] and by the exposure to new infectious agents [30–33].

Another hypothesis relates to climate changes affecting Europe during the period of the Neanderthal demise [34–37]. At the time of Neanderthal differentiation, Europe was characterized by a particular environment that underwent large climatic fluctuations, some of which were of a great magnitude with potential consequences for the expansion and/or reduction, and fragmentation of the Neanderthal metapopulation. Although Neanderthals had been coping with marked changes in climate and an associated turn-over in available food resources for almost 200,000 yrs [38,39], they failed to survive after the arrival of new hunter-gatherers, *Homo sapiens*.

All of these hypotheses, however, share the weakness of a much overlooked process of Neanderthal demography in its interaction with the changing environment. For instance, a small population size could have facilitated the replacement or the absorption of Neanderthal by *Homo sapiens*. Due to the lack of data, very little is known about the demography of past Neanderthal populations. Recent paleo-genetic studies have however estimated [23,40–45] the effective population size (index of genetic variability and not the census size). In spite of the fact that researchers agree on the “small size” of the Neanderthal population [2], its precise and accurate estimation remains difficult. Attempts on the basis of demographic modeling applied to Neanderthals proposed for the entire Neanderthal population (European and Asian) a maximum number of 70,000 individuals [46].

In this paper we are interested in understanding “how” Neanderthal disappeared. We explored qualitatively the possible cause of the Neanderthal population demise in terms of demographic changes, involving above all a reduction in its size. In the absence of palaeodemographic data regarding Neanderthal populations, we used demographic models to search for what values of demographic parameters could have maintained a demographically stable population. In a second step, we altered these values to quantify the necessary change in demographic parameters leading Neanderthals to extinction over a period of 10,000, 6,000 and 4,000 yrs *i*.*e*. within a time frame compatible with the known history of modern humans in Europe. In order to make our model more likely, the demographic parameters used are not stable over such a long time, but they change stochastically every year. In particular, we focused on the effect of a fertility reduction for primiparous females known in large mammals to be one of the first demographic rates affected by environmental variation (see [47–49]). Then we also examined the effects of reduced survival rates of different age-classes on extinction probability and time to demise. We started by projecting the effect of a reduction in survival of the youngest children, and finally studied two catastrophic scenarios: the situation of an epidemic and a war scenario, both of which would affect survival rates of adult individuals.

### Modeling Neanderthal population dynamics

To study how Neanderthals disappeared, we modeled their population dynamics with stochastic, age-structured matrix models [50,51]. This is a female-oriented model, where the demographic rates of males are supposed to mirror those of females. We also assumed that males are not a limiting factor for female reproduction, which is generally the case among polygynous species [48,52]. An important characteristic of long-lived species, *i*.*e*. species with a long life-expectancy, is the marked age-structure of its demographic rates [53]. For instance, populations of *Homo sapiens* [54], apes [52], mammalian large herbivores [49] and carnivores [55,56] or seabirds [57] all show a strong age-specific pattern of survival, with low survival rates during the juvenile stages, high survival of prime-aged individuals, and decreasing survival rates once the onset of senescence is reached (see [53] for a review). In the case of Neanderthals, we defined survival rates (*Ф*) for 5 age-classes: less than 1 y.o. (infant stage), from 2 to 15 y.o. (childhood), from 16 to 18 y.o. (sub-adults), from 19 to 29 y.o. (prime-aged adults) and over 30 y.o. (old). In this latter age group we find the maximum longevity [46]. We know that the longevity of Neanderthals could have been quite extensive [58–60] but, because of menopause, we assumed that the contribution of older individuals to the population growth rate was negligible and would not change our results while increasing the matrix dimension, and hence the calculation time. We set the earliest age for first reproduction of women Neanderthals to 18 y.o. Like survival rates, fertility varied with age, being lower for women aged between 18 and 20 y.o (primiparous) and higher for women between 21 and 30 y.o. (see below for details).

**Fig 1:**
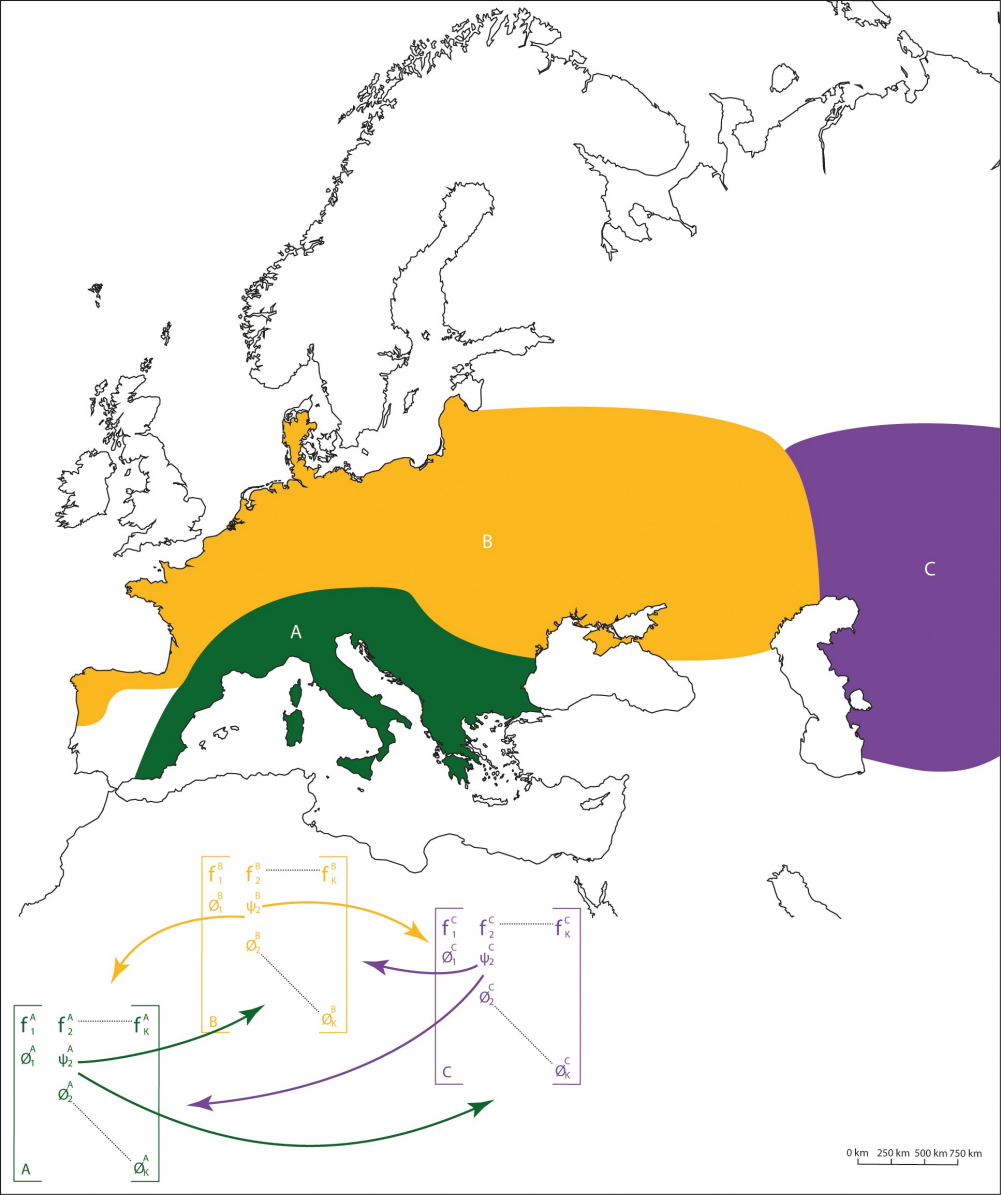
Spatial distribution and location of the 3 Neanderthal subpopulations. Southern Europe (labeled A in green), Northern Europe (labelled B in yellow), and Eastern Europe (labeled C in purple) according to [61]. The full demographic model we used to simulate Neanderthal population dynamics was composed of three sub-models corresponding to each of the identified sub-populations. We included a migration parameter (noted ψ) to allow for individuals to move from a sub-population to another.

We accounted for the spatial-structure of the European Neanderthal populations as revealed by recent genetic analyses [61]. We considered three discrete subpopulations labeled from West to East A, B and C (Fig 1) allowing for movements of individuals and for different demographic rates among subpopulations.

In our models, only individuals aged between 16 and 18 y.o., could migrate from one subpopulation to another. The rate of migrating Neanderthals varies among the three subpopulations and is asymmetric, immigration being different from emigration for a given subpopulation [61]. This movement pattern reflects the environmental and social constraints associated with the colonization of Western Europe by modern humans from the East [62,63].

## Material and methods

Although the size of the Neanderthal population is not known accurately, we have started with an optimistic initial population size of 35,000 females corresponding to the estimated population size [46] divided by two, hence assuming an even sex-ratio at the population level.

We then used the Leslie matrix to analyze in detail (by age) the role of demographic parameters (fertility, survival and migration) over time in three geographical regions.

We used the recurrence Eq [1] to simulate the spatio-temporal variation in population size over a 10,000 year period (*t* = {1, …, 10000}) with a post-breeding Leslie matrix.

$$N_{t+1}=L_{t}.N_{t},$$ [1]

where **N** is the population vector and **L** is the transition matrix. At each time-step *t*, all demographic rates of **L**_t_ were drawn at random in age and subpopulation-specific density probability function (Table 1) using beta distributions for survival (*Ф*) and dispersal (*ψ*) rates, and a Poisson distribution for the number of female offspring per fecund females (*f*) [64]. The time span of the simulation corresponded to the elapsed time between the maximum population size estimates and the current estimated time of the last Neanderthal site occurrence [65]. This time span is less than 10,000 yrs [65].

**Table 1:** Demographic parameters entered in the stochastic Leslie matrix (mean and standard errors) to project population size of Neanderthals according to different scenarii of Neanderthal time of extinction in Western Europe. In order to make our model more likely, the demographic parameters used are not stable over such a long time, but they change stochastically every year.

Subpopulation	Demographic parameter	Survival rate	Demise in 10,000 yrs	Demise in 6,000 yrs	Demise in 4,000 yrs
A, B and C	Infant survival	0.720 ± 0.10	=	=	=
	Sub-adult survival	0.955 ± 0.05	=	=	=
	Prime age survival	0.970 ± 0.045	=	=	=
	Adult survival	0.990± 0.025	=	=	=
	Old survival	0.980 ± 0.09	=	=	=
A	Primiparous reproduction	0.1415± 0.055	0.1376 ± 0.055	0.1350 ± 0.055	0.1300 ± 0.055
	Adult reproduction	0.2700 ± 0.055	=	=	=
B	Primiparous reproduction	0.1415± 0.055	0.1376 ± 0.055	0.1350 ± 0.055	0.1300 ± 0.055
	Adult reproduction	0.2700 ± 0.055	=	=	=
C	Primiparous reproduction	0.1700 ± 0.10	0.1376 ± 0.055	0.1350 ± 0.055	0.1300 ± 0.055
	Adult reproduction	0.2700 ± 0.055	=	=	=
A → B	Emigration	0.0010 ± 0.005	=	=	=
B → A	Emigration	0.0020 ± 0.005	=	=	=
A → C	Emigration	0.0001 ± 0.005	=	=	=
C → A	Emigration	0.0005 ± 0.005	=	=	=
B → C	Emigration	0.0010 ± 0.005	=	=	=
C → B	Emigration	0.0050 ± 0.005	=	=	=

**Fig 2:**
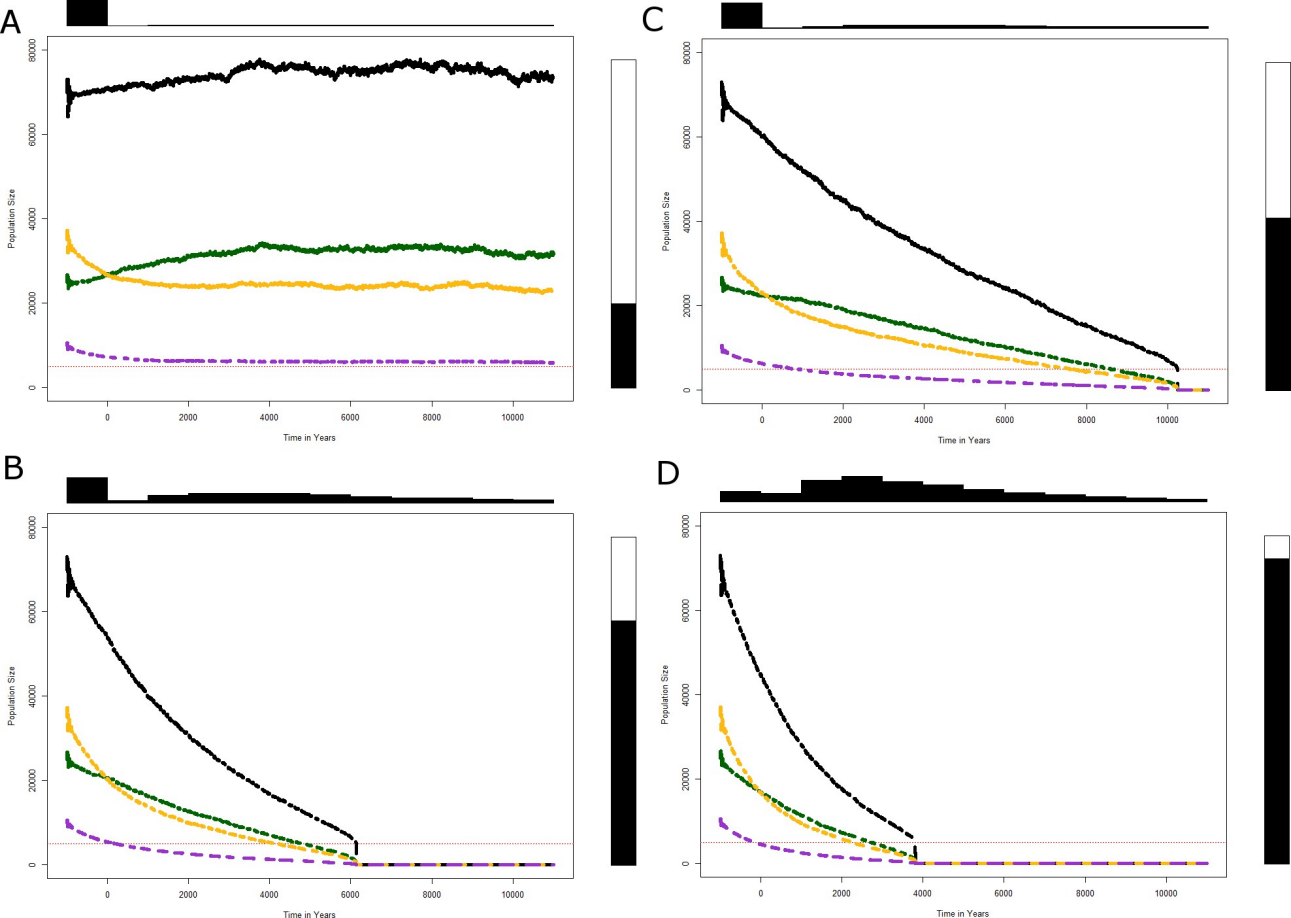
Simulated population trajectories of the Neanderthals over 10,000 yrs. Lines color correspond to the three subpopulations of Neanderthals in Europe (see Fig 1: subpopulation A in green, B in yellow and C in purple) and in black to total population. Dotted red line shows the MVP (minimum viable population). The top-panel histogram displays the distribution of the time at extinction of the whole Neanderthal population. Right panel gives the proportion of simulated trajectories that hit the threshold population size of 5,000 under which the population was considered as extinct, e.g. the quasi-extinction probability. We present results of median of the 10,000 simulations for scenarios where the overall Neanderthal population never goes extinct (**Fig 2A** Parameters used in the simulation are shown in Table 1 “Survival”), disappears in 10,000 yrs (**Fig 2B** Parameters used in the simulation are shown in Table 1 “Demise in 10,000 yrs”), 6,000 yrs (**Fig 2C** Parameters used in the simulation are shown in Table 1 “Demise in 6,000 yrs”) and 4,000 yrs (**Fig 2D** Parameters used in the simulation are shown in Table 1 “Demise in 4,000 yrs”).

Regarding the migration flows between three Neanderthal subpopulations in Europe, we first assumed a very low population density for Eastern European Neanderthals (subpopulation C), which is confirmed by the extremely high rate of endogamy of Neanderthals reported in Eastern Europe [44,45]. The young individuals of subpopulation C are more likely to find a partner by migrating to the West and South. Although very low (set to a rate of 0.005), this dispersal rate led to a rapid erosion of the size of subpopulation C (see Fig 2A, 2B, 2C and 2D). In our model, the individuals aged between 15 and 18 of the Northern subpopulation B could migrate south and contribute to the increase of the Southern subpopulation A. As testified by the archaeological data, this latter group was the last subpopulation to disappear [65,66]. Some authors even regarded Southern Europe to be a Neanderthal refuge zone [35,67], but this hypothesis has recently been questioned [68,69].

The dimension of the transition matrix **L**_**t**_ was of 105 rows by 105 columns (i.e. 35 age-classes, from 1 to 34 and a class for > 35, for each of the 3 subpopulations). For each completed run, we calculated the time to extinction and the quasi-extinction probability across the 10,000 simulations. We considered a subpopulation or the whole population as extinct when its size felt below 5,000 individuals. According to ecologicaly studies [70,71], the critical size or “minimum viable population” (MVP) is the point of no return beyond which extinction will certainly occur. Given that the demographic parameters such as, for example, survival, fertility rates, and population structure, were not precisely known for Neanderthal populations because of lack of life-table data, we first set the distribution of model parameters (average and dispersion) based on the median demographic rates observed in populations of modern humans with a hunter-gatherer lifestyle, and in populations of large apes extracted from the literature [72–76]. It should be noted that the model is such that the initial demographic parameters (the age distribution of the population, the number of individuals) did not affect the result, since after a few generations the structure is determined by the fertility, survival and migration rates. We monitored time-specific abundance for the three subpopulations (A, B, and C), as well as for the whole population. In the following, we report the median, and 0.025 and 0.975 percentiles for each model output. All simulations and computations were performed using the R software [77].

Based on the demographic parameters we retrieved from the literature, the Neanderthal population was found to be stable (population growth rate λ = 1). Second, holding everything else constant, we reduced the fertility of primiparous women starting from a value of 0.1415. In long-living species like hominids, the population growth rate is much less sensitive to variation in recruitment parameters like juvenile survival than to variation in survival of adults [78]. Consequently, natural selection shaped life-histories of long-living species with a high and constant adult survival but a highly variable recruitment, a phenomenon known as environmental canalization [79]. Such a sequence in the relative variation of demographic rates in space and time has been reported repeatedly in other large mammal populations [48,49], including human populations [80,81], in response to environmental adversity like increasing population density or decreasing food resources. Assuming a similar functioning of Neanderthal population dynamics, we decreased the prime age fertility rates until the simulated time at extinction fell within the confidence limits of the observed time of extinction of Neanderthals. Since adult female survival is the most resilient demographic parameter to environmental perturbation, we kept it unchanged. For each explored scenario, “Survival”, “Demise in 10,000 yrs”, “Demise in 6,000 yrs” and “Demise in 4,000 yrs”, we replicated the simulations of the population trajectories of Neanderthals 10,000 times. In a second step we kept all the parameters constant and lowered the survival of the youngest child until reaching the extinction of the whole population and finally we reduced survival rates of adult individuals to study two catastrophic scenarios: the situation of an epidemic and a war scenario.

## Results

We first used average demographic rates extracted from the literature on hunter-gatherer humans and great apes as the average for a random draw. These rates were converted into annual rates (Table 1 column “Survival”) to parameterize the projection matrix and then to simulate population trajectories over a time period of 10,000 yrs (Fig 2A).

After few iterations the model converged to nearly asymptotic dynamics, and the average of the 10,000 simulated trajectories of the total Neanderthal population size and of the three subpopulations (A, B and C) stayed quite stable with a generation time of 25 yrs. With these demographic parameter values, the extinction probability over the 10,000 yrs was relatively low (P = 0.2) for the whole population and for the Westernmost subpopulations (A and B). The extinction probability for the Eastern subpopulation C, which happens to be the smallest too, was higher, reaching P = 0.6 (Table 2, column “Survival”).

**Table 2:** Extinction probability and average time of extinction for the overall Neanderthal population and for each of the 3 subpopulations. We report the outcome of 10,000 simulated trajectories and the decrease in reproduction rate of primiparous women required for the extinction of Neanderthals in 10,000, 6,000 and 4,000 yrs.

	Population	Survival rate	Demise in 10,000 yrs	Demise in 6,000 yrs	Demise in 4,000 yrs
Primiparous reproduction rate	A, B and C	0.1415	0.1376	0.135	0.13
Extinction probability	A	0.28	0.55	0.76	0.94
	B	0.29	0.57	0.77	0.94
	C	0.60	0.83	0.94	0.99
	Total	0.26	0.53	0.75	0.93
Average time to extinction	A		11,240	7,132	4,809
	B		11,238	7,132	4,804
	C		10,661	6,594	4,341
	Total		11,242	7,134	4,811

We then successively decreased the value for the fertility rates of young females, initially set at 0.1415 (Table 1 column “Survival”) in each subpopulation A, B, and C. We found that by slightly altering the reproduction of young females to 0.1376 (-2.7%) in each subpopulation, the average total population size of Neanderthals fell below the threshold of 5,000 individuals within less than 10,000 yrs (Fig 2B). We tabulated the average time to extinction and probability of extinction for this model in Table 2 column “Demise in 10,000 yrs”. As expected from the imposed changes in demographic parameters, subpopulations did not become extinct at the same time, with the easternmost population (C) collapsing first, followed by the Northern subpopulation (B) and then the Southern subpopulation (A). We obtained comparable but more dramatic results when the fertility rate of younger women was further reduced to 0.1345 (-5%: Fig 2C, Table 2 column "Demise in 6,000 yrs") and even more when lowered to 0.1300 (-8%: Fig 2D, Table 2 column "Demise in 4,000 yrs"). Note that the models we proposed differ in the fertility rate of the younger females only as it adopted different values for each subpopulation each year. The difference between "stable" and "demise" fertility values is minimal, but large enough to bring about the disappearance of the Neanderthals over a period of between 10,000 and 4,000 yrs, without the need to take into account changes in survival rates.

**Fig 3:**
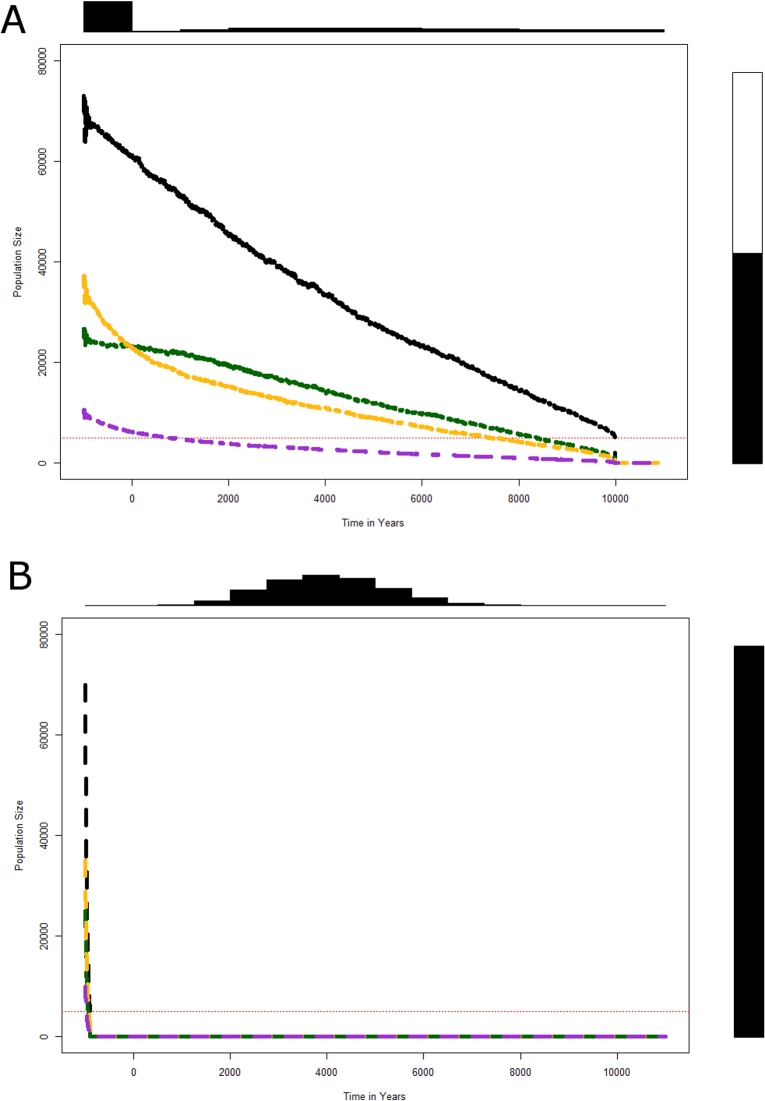
Simulated trajectories for the Neanderthals overall population and for the 3 sub-populations with reduced survival. Parameters used in the simulation are shown in Table 1 “Survival”, reducing young infants survival by 0.4% (Fig 3A) or reducing adult survival by 10% (Fig 3B).

Then we analyzed the effect of the reduction in survival (and consequently the increase in mortality) of infants (<1 y.o.). Starting again from the values of the demographic stability of the population (Table 1 column “Survival”), we decreased the survival rate and found that a decrease of 5% in the survival rate (0.6850) every year, holding the other parameters unchanged, led to an extinction of the population in 20 yrs. For the time to extinction of Neanderthals to match 10,000 yrs, we had to reduce survival by only 0.4% (0.7171) (Fig 3A), while a reduction by 1% (0.7128) causes an extinction in almost 6,000 yrs. We finally explored the possible effects of a disease transmitted by *sapiens* or of a conflict that would have substantially affected survival rates of adults: from the parameters of the "Survival" model reducing adult survival by 10% (keeping all other parameters identical), the whole Neanderthal population became extinct extremely fast (Fig 3B).

## Discussion

The main difficulty when working with Neanderthals is the scarcity of empirical data to reliably test the several hypotheses that could account for their disappearance. From a demographic point of view, we only know that Neanderthals existed and disappeared at some point in the past, but we do not know why they disappeared and how long it took for them to become extinct. Either a single or several events might have come into play. These constraints led us to formulate very simple models and to explore the expected dynamics with plausible values of fertility, survival and migration for human populations. For instance, we disregarded a combination of demographic parameters leading to very low extinction probability because this clearly did not occur. For the few empirical data we have, like population size or the time of extinction, the accuracy of the estimates is very low at best.

Nevertheless, our aim here is not to evaluate accurate estimates of demographic parameters, but to explore the range of possible values that can generate a decreasing trend in Neanderthal populations.

We showed that, in the long run, a slight change in the fertility rate of younger females could have had a dramatic impact on the growth rate of the Neanderthal metapopulation and thus on its long-term survival, in agreement with the observed extinction of Neanderthals within a 10,000, 6,000 or a 4,000 years period. Our modelling suggests that it is not necessary to explain the decrease in size of the Neanderthal population on the basis of catastrophic causes (diseases, extreme climatic events, and disasters such as volcanic eruptions. . . .) or even of the direct or indirect intervention of *sapiens*.

By lowering the average fertility rate from 0.141 to 0.137 for "primiparous reproduction", the population dynamics of Neanderthals switches from a stable or sometimes increasing population to a decreasing population in time which, on the average, eventually dies out over a period of 10,000 yrs,. If the average fertility rate is slightly reduced to 0.135 (or 0.130), this disappearance, on the average, is attained in just 6,000 yrs (or 4,000 yrs). This shows that it only takes a slight decrease in resources over a period of some years to cause a decrease in fertility [82]. It is interesting to note that we have modified primiparous fertility only, therefore focusing on a single class of individuals which comprises 10% of the overall female population (according to the stable age-structure of the model). If our modeling exploration cannot identify the origin of a decrease in fertility of young women, at least putative mechanisms can be put forward: food stress. Because the amount of stored body fat influences fertility in women [82] a decline in resources (caused by climate degradation or competition with *sapiens*) may affect fertility mostly for young women giving birth for the first time. This hypothesis is consistent with the analyzes of exploitation of the bones of fauna carried out in the South of France [83] that indicate that Neanderthals could have been nutritionally stressed.

Besides, although our study is focused on women, disappearance of males could be linked to women fertility. If little is known about the contribution of Neanderthal women to the retrieval of food resources for the group [84], the male contribution was crucial for the group survival. A significant loss of men due to inter-individual conflicts or during hunting activities would have been of great importance for their physical condition, and hence for female Neanderthal fertility.

Neanderthal reproductive decline could be amplified by *Homo sapiens*. Neanderthals and *Homo sapiens* experienced some hybridation in Central Asia and in Western Siberia [85–88] and on the European continent, as suggested by anthropological [89–91] and genetic evidence [85,86,92]. This hybridation, although important for *sapiens* allowing the introgression of several useful alleles (see [93] for a review; [45,85]) concerned however a very small number of individuals, since one individual is estimated for every 300 [94] or 250 yrs [95]. Indeed the genetic comparison of the Y chromosome between present-day humans and a Neanderthal of El Sidron [96] suggests that some mutations present in Neanderthals could have caused infertility problems in male hybrids. Such hybrids with less fertility may have contributed to a slight decrease fertility rate [96,97] in Neanderthal population, whereas in *sapiens* population their high number would have made crossings large enough to lead to the suppression of these deleterious alleles.

In agreement with a previous publication [61], we emphasize that we considered a subdivision of Neanderthals among three populations, but given the low Neanderthal population density, we could suppose a stronger fragmentation. Indeed, on the one hand, the three geographical areas considered are wide and heterogeneous from the environmental point of view and, on the other hand, the way of life of Neanderthals as hunter-gatherers corresponds to a clan structure of interconnected individuals [98]. Therefore fragmentation of the metapopulation was probably greater, causing a postponement of their demise [99,100]. By reinforcing demographically the weakest populations on the verge of extinction, the migration process decreased the probability of extinction of the overall metapopulation dramatically, as the theory predicts [101]. Obviously, in the absence of migration, the disappearance of the Neanderthals would have been even more rapid and likely [99,100].

The effects of decreased survival on the extinction probability and time to extinction are considerable as expected for long-living organisms: a decline of less than 1.5% in survival for the youngest children leads to rapid extinction (less than 2,000 yrs), while a reduction of survival rate as small as 0.4% provokes an extinction time of 10,000 years. Another important result of our model is that the disappearance of Neanderthals caused by diseases (infectious and other) contracted by contact with *sapiens* and leading to a high mortality rate leads to very rapid and sudden extinction. Assuming for instance an infant survival reduced by 10% [102–105]: the demise of Neanderthals would have been much faster than what the archeological records currently suggest. Moreover, owing to the very low Neanderthal density, this hypothesis could account for local disappearances of Neanderthal groups and could not lead to complete demise of the entire population [106]. Similarly, due to the low density of Neanderthals, higher mortality resulting from violence between the two populations could only explain a local decrease in size and extinction, but it would not be applicable to the entire geographical space occupied by Neanderthals. Nevertheless, when exploring this hypothesis, from initial value by reducing adult survival by 10% (keeping all other parameters identical), the whole of the Neanderthal population became suddenly extinct (Fig 3B).

Our results lead us to the conclusion that the size of the Neanderthal population could have slowly and gradually decreased over time and that when it was already small and began to decline, *Homo sapiens* may well have simply taken advantage of an already low density of Neanderthals in order to settle into Europe. As proposed for the Iberian region [107] a low growth rate can be at the origin of Neanderthal disappearance. Our model can make possible to better understand Neanderthal demise at the level of the entire territory and to identify the role of each demographic parameters in this process.

Modeling is shown to be a useful tool for answering the question concerning the disappearance of this population on such a huge geographical space as Europe, Asia and the Near East and at a time that is not yet exactly known.
